# Characterizing public participation in Prescription Drug User Fee Act reauthorization, 2010–2021

**DOI:** 10.1093/haschl/qxag169

**Published:** 2026-07-04

**Authors:** Therese J Ziaks, Asuka Koda, Reshma Ramachandran, Joseph S Ross, Jason L Schwartz

**Affiliations:** Yale Collaboration for Regulatory Rigor, Integrity, and Transparency, Yale School of Medicine, New Haven, CT 06510, United States; Yale University, New Haven, CT 06510, United States; Yale Collaboration for Regulatory Rigor, Integrity, and Transparency, Yale School of Medicine, New Haven, CT 06510, United States; Section of General Internal Medicine, Department of Internal Medicine, Yale School of Medicine, New Haven, CT 06510, United States; Yale Collaboration for Regulatory Rigor, Integrity, and Transparency, Yale School of Medicine, New Haven, CT 06510, United States; Section of General Internal Medicine, Department of Internal Medicine, Yale School of Medicine, New Haven, CT 06510, United States; Department of Health Policy and Management, Yale School of Public Health, New Haven, CT 06510, United States; Yale Collaboration for Regulatory Rigor, Integrity, and Transparency, Yale School of Medicine, New Haven, CT 06510, United States; Department of Health Policy and Management, Yale School of Public Health, New Haven, CT 06510, United States

**Keywords:** PDUFA, user fees, Prescription Drug User Fee Act

## Introduction

Since 1992, a substantial portion of the US Food and Drug Administration (FDA) budget has been supported by industry user fees, in accordance with the Prescription Drug User Fee Act (PDUFA) and other related legislation.^1^ In fiscal year (FY) 2025, user fees comprised over 70% of FDA's total budget for human drugs.^2^ The PDUFA requires reauthorization every 5 years, a process that includes closed-door negotiations between FDA and the pharmaceutical industry regarding the use of user fee funds, culminating in a commitment letter agreed to by both parties and transmitted to US Congress.

Since 2003, a parallel series of meetings between FDA and public stakeholders have been held during the negotiation period, as required by statute.^1^ These are intended to provide opportunities for the public, including patient and consumer advocacy groups, academic research groups, professional organizations, and unaffiliated individuals, to share perspectives with FDA that can inform the agency's concurrent negotiations with industry.

Examining the content of public stakeholder meetings held as part of recent PDUFA reauthorization may provide insights regarding priorities and concerns of patient and consumer stakeholders and the degree to which PDUFA itself—through the commitment letters negotiated with industry and the ensuing legislation passed by Congress—reflects those public stakeholder perspectives.

## Methods

Minutes for public stakeholder meetings during the 3 most recent publicly posted PDUFA cycles—V (2013–2017), VI (2018–2022), and VII (2023–2027)—were identified from FDA's website. Summary information for each meeting, including dates, frequency, and participants (FDA staff and public stakeholders), were collected. Participants and organizations in attendance were assigned to categories reflecting their professional identity and focus using the nonprofit organization database Guidestar.^3^ Websites of PDUFA VII meeting attendees or organizations were reviewed to identify any financial, leadership, or collaborative relationships with the pharmaceutical industry. Meeting discussion topics and recommendations recorded in the meeting minutes, including those offered directly by public stakeholders and by the FDA, based on the interests of public participants and topics of the concurrent FDA–industry meetings, were extracted and organized by the major elements and goals of PDUFA and the FDA activities that it supports. Minutes were reviewed and coded using a grounded theory approach by 2 research team members (T.J.Z. and A.K.); discrepancies were resolved by a third member (J.L.S.). Descriptive statistics were calculated using Excel. The study followed the STROBE (Strengthening the Reporting of Observational Studies in Epidemiology) reporting guidelines; institutional review board approval and participant informed consent were not required for this review because it used only publicly available information.

## Results

We identified 22 public meetings held during these 3 PDUFA cycle negotiations with publicly available minutes: 10 meetings for PDUFA V, 6 meetings for PDUFA VI, and 6 meetings for PDUFA VII. Among 74 registered organizations for PDUFA V meetings, 31 (41.9%) represented patient advocacy and disease organizations, 15 (20.3%) professional organizations, and 7 (9.5%) other consumer groups (ie, organizations without a focus on specific diseases) (Table 1); similar patterns were observed for the 50 registered organizations for PDUFA VI meetings (30 [60.0%], 5 [10.0%], and 7 [14.0%], respectively), as well as for the 55 registered organizations for PDUFA VII meetings (29 [52.7%], 7 [12.7%], and 7 [12.7%], respectively).

**Table 1:** Categorization of public stakeholder organizations represented at PDUFA V–VII reauthorization meetings.

	Patient advocacy and disease organizations, No. (%)	Academic institutions, No. (%)	Other consumer groups,^a^ No. (%)	Professional organizations, No. (%)	Other (patients, unknown), No. (%)	Payer, No. (%)	Other industry, No. (%)	Coalition, No. (%)	Think tanks, No. (%)	Total, No.
PDUFA V	31 (41.9)	2 (2.7)	7 (9.5)	15 (20.3)	2 (2.7)	1 (1.4)	6 (8.1)	5 (6.8)	5 (6.8)	74
PDUFA VI	30 (60.0)	1 (2.0)	7 (14.0)	5 (10.0)	3 (6.0)	0 (0)	0 (0)	2 (4.0)	2 (4.0)	50
PDUFA VII	29 (52.7)	4 (7.3)	7 (12.7)	7 (12.7)	1 (1.8)	0 (0)	0 (0)	6 (10.9)	1 (1.8)	55

Abbreviation: PDUFA, Prescription Drug User Fee Act.

^a^Consumer groups are organizations that advocate for the consumer perspective as a part of the regulatory process, which excludes patient advocacy, health professional organizations, academic institutions, and other individuals.

During 10 PDUFA V meetings, speed (*n* = 8), regulatory flexibility (*n* = 7), postmarket surveillance (*n* = 7), FDA–industry interaction (*n* = 7), public engagement (*n* = 7), and “other” (*n* = 9) were common themes, while staffing/resource discussions were less common (*n* = 1). Topics mentioned in the “other” category included transparency, labeling, information technology, drug shortages, advisory committees, off-label prescribing, generic drug user fees, comparative effectiveness research, direct-to-consumer advertising, the Reagan-Udall Foundation, and gene therapy. In contrast, during 6 PDUFA VI meetings, speed (*n* = 2), FDA–industry interaction (*n* = 2), and other (*n* = 0) were less common while regulatory flexibility (*n* = 6), postmarket surveillance (*n* = 4), and public engagement (*n* = 4) were common themes. Of 6 PDUFA VII meetings, staffing (*n* = 4), postmarket surveillance (*n* = 3), regulatory flexibility (*n* = 5), and public engagement (*n* = 5) discussions were more common, while speed (*n* = 1) and other (*n* = 2) discussions were less common (Appendix).

Of the 22 total meetings, regulatory flexibility was the most discussed topic (*n* = 18), followed by public engagement (*n* = 16), postmarket surveillance (*n* = 14), speed (*n* = 11), FDA–industry interaction (*n* = 11), other (*n* = 11), and staffing and resources (*n* = 9). Of 54 PDUFA VII public stakeholder meeting attendees (1 excluded), 45 (83.3%) had pharmaceutical industry ties.

## Discussion

The PDUFA public stakeholder meetings provide an opportunity for perspectives outside of the pharmaceutical industry to inform FDA–industry negotiations central to the eventual legislation and FDA activities it funds. In these meetings, patient advocacy organizations had the highest participation of public stakeholders, pharmaceutical industry ties were common, and regulatory flexibility and public engagement were the most common topics. Payers were infrequent attendees at the 3 reauthorization cycles examined, with only 1 payer attending meetings preceding PDUFA V.

Due to limited public information provided regarding the details of FDA–industry negotiations while they are underway, these parallel series of stakeholder meetings primarily function as listening sessions, organized around presentations by FDA staff about programs and activities in which stakeholders have expressed interest. Future research could evaluate the degree to which this structure serves the intended purposes of these meetings and meaningfully contributes to the FDA–industry negotiations happening concurrently.

Study limitations include the lack of publicly available meeting transcripts and limited detail within meeting minutes regarding stakeholders’ comments. Minutes for the initial “Kickoff Meeting” held at the start of each reauthorization cycle and open to both industry and public stakeholders were not publicly posted and therefore not available for analysis. Coding was subject to reviewer interpretation.

As PDUFA VIII reauthorization continues, understanding public stakeholder participation in previous reauthorization cycles provides insight on potential areas for strengthening this process in the future, such as FDA facilitating increased participation by stakeholders without ties to the pharmaceutical industry, and enhancing its contributions to the overall user fee program.

## Supplementary Material

qxag169_Supplementary_Data

## References

[1] Ziaks TJ, Schwartz JL, Ross JS, Ramachandran R. Reforming the prescription drug user fee program. N Engl J Med. 2025;393(8):734–736. 10.1056/NEJMp2507648 40834320

[2] US Food and Drug Administration Office of the Commissioner . FDA at a glance. 2026. Accessed March 1, 2026. https://www.fda.gov/media/154548/download

[3] Guidestar . Home page. Accessed December 1, 2025. https://www.guidestar.org/

